# Odontogenic tumors and giant cell lesions of jaws - a nine year study

**DOI:** 10.1186/1477-7819-9-68

**Published:** 2011-07-05

**Authors:** Surekha Venkata Mullapudi, Uday Kumar Putcha, Sesikeran Boindala

**Affiliations:** 1 PATHOLOGY DIVISION National Institute of Nutrition (Indian Council of Medical Research), Hyderabad Andhra Pradesh, India

## Abstract

**Objectives:**

A definite geographic variation has been observed in the frequency of odontogenic tumors and giant cell lesions of the jaws reported from different parts of the world. However, there are a few studies on these lesions, especially giant cell lesions, reported from India. Hence, this study was designed to provide a demographic data on the odontogenic tumors and giant cell lesions reported from our institute located in the city of Hyderabad. Hyderabad is the capital city of the southern state of Andhra Pradesh in India. A retrospective analysis of odontogenic tumors and giant cell lesions of jaws reported in our institute between the years 2000 and 2009 was done and this data was compared with previous reports from different parts of the world and India.

**Methods:**

Biopsies of the lesions received between the years 2000 and 2009 were reviewed and patient's history, clinical, radiological and histopathological characteristics were analyzed.

**Results:**

A total of 77 biopsies were received during the nine year study period. These lesions were more frequently seen in the males, in a younger age group and showed a predilection for the mandible. Most of them presented as radiolucent, slow growing and painless lesions. Ameloblastomas (71.4%) constituted the majority of odontogenic tumors while central giant cell granulomas (7.8%) constituted the majority of giant cell lesions.

**Conclusion:**

These lesions showed a definite geographic variation with ameloblastomas being the most common odontogenic tumors and odontomas being relatively rarer lesions in our region.

## Introduction

The oro-facial region including the jaw bones, maxilla and mandible, is a site for a multitude of neoplastic conditions [1].

Odontogenic tumours are lesions derived from epithelial, ectomesenchymal and/or mesenchymal elements that are a part of the tooth forming apparatus. The majority of these tumors occur intraosseously within the maxillofacial skeleton, while extraosseous odontogenic tumors occur nearly always in the tooth-bearing mucosa [2]. Due to their specific structure and location they have been identified and classified by pathologists into a separate group, differing in histogenesis, biology, clinical manifestations and radiological signs from other tumors developing in the oral cavity and facial bones [3].

Giant cell lesions of the jaws are benign, tumor-like lesions affecting the jaws but also occurring in other bones and soft tissues. Their biologic behavior in the jaws is identical to that in the long bones and is unrelated to patient's age and size of the lesion [4]. They consist of multinucleated giant cells in a background of fibrous connective tissue with abundant spindle-shaped mononucleated cells [5].

Till date several retrospective studies have been carried out in different parts of the world like Africa, Asia, Europe and America which show a definite geographic variation in the relative frequency, site and histologic type of these lesions [1-5,10-35]. Very few studies have been reported from Asia, especially from the Indian subcontinent [6-8,36-38]. Although these few studies from India demonstrate the frequency of the various odontogenic tumors, reports on giant cell lesions are however very scarce. Hence, the present 9 years retrospective study was conducted to analyze the frequency, clinical presentation, site and character of the odontogenic tumors and giant cell lesions reported in our institute from the city of Hyderabad located in the south Indian state of Andhra Pradesh.

The aims of our present study were to retrospectively analyze these varied lesions clinico-pathologically and to compare this data with the data from different parts of the country and also the world.

## Materials and methods

A retrospective study was performed on 77 patients who underwent surgery for jaw lesions between the years 2000 and 2009. Data was retrieved from case notes, radiographs and histopathology results reported in the department of Pathology of National Institute of Nutrition, Hyderabad, India. Patient's history, clinical findings, radiological and histopathological characteristics were analyzed. Hematoxylin and eosin (H&E) stain was used on sections of buffered formalin fixed tissues.

## Results

Records of a total of 77 patients who presented with jaw swellings between the years 2000 and 2009 were retrieved and the tumors were classified according to the World Health Organization 2005 classification of odontogenic tumors [2]. Of the 77 tumors reported, all were benign lesions.

**Figure 1:**
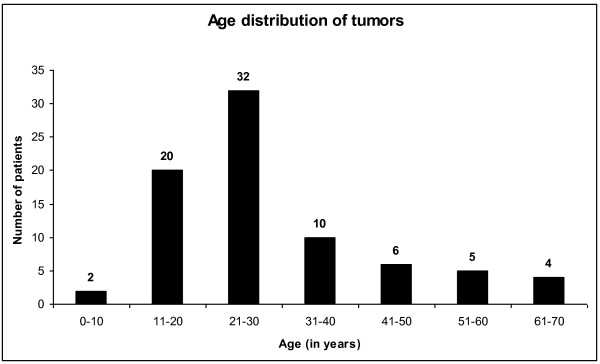
**Shows the age distribution of patients with different odontogenic tumors and giant cell lesions of the jaws**.

These lesions were detected in both sexes, with males comprising 53.2% (N = 41) of all the 77 patients seen and the rest 46.8% (N = 36) being females. The male:female ratio was 1.1:1.0. The mean age of patients at the time of presentation was 25 years with most (N = 32) being in the age group of 21 to 30 years (Figure 1). Sixty-three (81.8%) out of the 77 tumors were encountered in the mandible with the overall mandible:maxilla ratio being 4.5:1.

Out of the 77 cases, ameloblastomas were the most common odontogenic tumors encountered (71.4%) followed by adenomatoid odontogenic tumors(8.5%), calcifying epithelial odontogenic tumors(7.1%), odontomas (4.5%), odontogenic myxomas(4.5%) and odontogenic fibromas(2.8%). Among the giant cell lesions, central giant cell granulomas constituted 7.8% of all the jaw lesions followed by aneurysmal bone cyst (1.2%).

Sixty-five patients presented with a swelling in one of the jaw bones. Majority of these jaw swellings (N = 62) presented with a slow growth while rapid growth was observed in 3 cases. The swellings were associated with pain in 6 cases while the history of pain in the remaining cases was unavailable in our study. Eleven cases were associated with impacted teeth.

Radiologically out of the 77 jaw lesions, majority (N = 59) were radiolucent lesions while only 6 were radiopaque. Radiological findings of the remaining 12 cases were unavailable for study. Most of the radiolucent lesions were multilocular while majority of the radiopaque lesions were diagnosed as odontomas on histology.

Histopathologic typing revealed that out of 77 lesions studied, majority (N = 70, 91%) were odontogenic tumors and the remaining (N = 7, 9%) were giant cell lesions. The histopathologic typing of the 77 odontogenic tumours and giant cell lesions of jaws seen in nine years at Hyderabad is shown in Table 1.

**Table 1:** Shows odontogenic tumors and giant cell lesions according to the WHO histological classification of odontogenic tumours, 2005.

Tumour	No. (%)
**A. Malignant tumours**	0 (0%)

**B. Benign Tumours**	77 (100%)
**ODONTOGENIC TUMOURS:**	70 (91%)
a)Odontogenic epithelium with mature, fibrous stroma without odontogenic ectomesenchyme.	
i) Ameloblastoma	50 (71.4%)
ii) Calcifying epithelial odontogenic tumour	05 (7.1%)
iii) Adenomatoid odontogenic tumour	06 (8.5%)
	
b)Odontogenic epithelium with odontogenic ectomesenchyme with or without hard tissue formation	
i)Ameloblastic fibroma	01 (1.4%)
ii)Odontoma	03 (4.3%)
	
c)Mesenchyme and/or odontogenic ectomesenchyme with or without odontogenic epithelium	
i)Odontogenic fibroma	02 (2.8%)
ii)Odontogenic myxoma	03 (4.3%)
	
1. **GIANT CELL LESIONS:**	
a) Bone related lesions	7 (09%)
i)Central giant cell granuloma	06 (7.8%)
ii)Aneurysmal bone cyst	01 (1.2%)

### Odontogenic tumors constituted 70 (91%) of the total 77 jaw lesions studied

**Figure 2:**
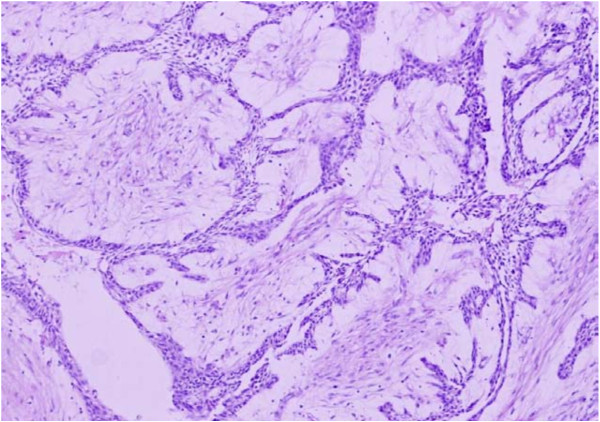
**Microphotograph of plexiform ameloblastoma**. H&E. 20×.

**Figure 3:**
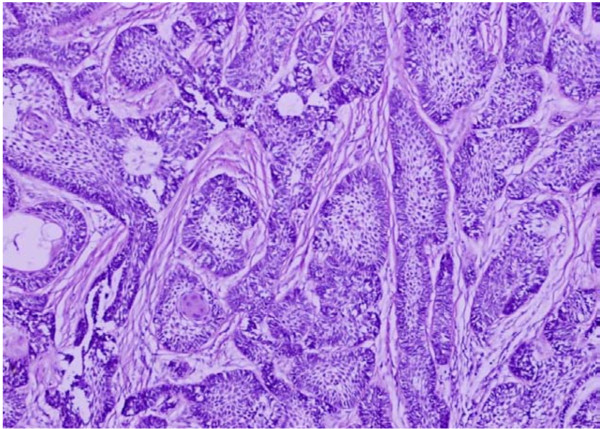
**Microphotograph of follicular ameloblastoma showing islands of odontogenic epithelium within sparsely cellular stroma**. H&E. 20×.

**Figure 4:**
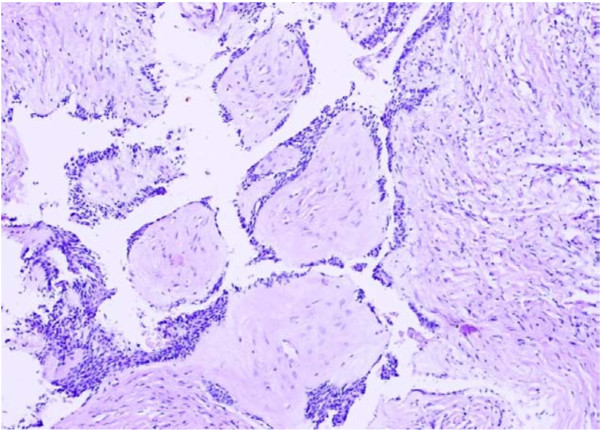
**Microphotograph of desmoplastic ameloblastoma showing islands of odontogenic epithelium in a desmoplastic stroma**. H&E. 20×.

Of the 70 odontogenic tumors studied, ameloblastomas (Figures 2, 3, 4) were the most frequently encountered lesions, constituting up to 71.4% (N = 50) of the tumors, with majority of these patients (N = 22, 44%) being in the age group of 21 to 30 years. These lesions occurred more in males (N = 28, 56%) than in females (N = 22, 44%) with 86% (N = 43) being mandibular lesions which were mostly (N = 36) observed to be multilocular on radiology. Table 2 shows the frequency of occurrence of the different histological types of ameloblastomas encountered in our study.

**Table 2:** Shows the different types and the frequencies of ameloblastomas.

Types of Ameloblastoma	No. (%)
1. Solid/Multicystic type	45 (90%)
	
a) Follicular	22 (48.9%)
b) Plexiform	18 (40.1%)
c) Acanthomatous	03 (6.6%)
d) Basaloid	02 (4.4%)
2. Desmoplastic	03 (6%)
3. Unicystic	02 (4%)

Total	50

**Figure 5:**
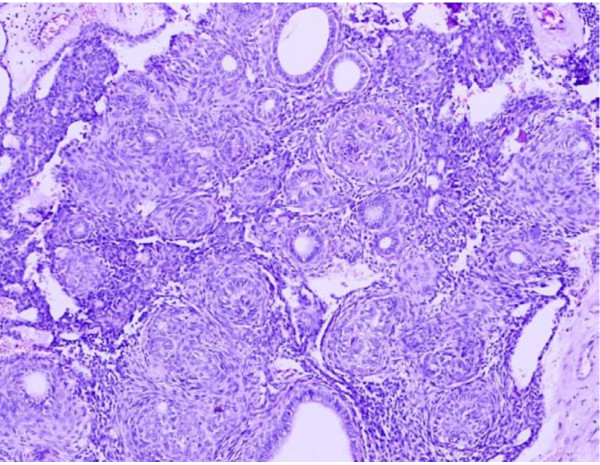
**Microphotograph of adenomatoid odontogenic tumor with mixed islands of odontogenic epithelium and columnar cells**. H&E. 20×.

The tumors next in frequency to ameloblastomas, and second in the series of odontogenic tumors were adenomatoid odontogenic tumors (Figure 5), constituting 8.5% (N = 6) of the 70 odontogenic tumors and with most of the patients presenting in the younger age group of 11 to 20 years. Females (N = 4, 66.6%) outnumbered the males in incidence and mandible (N = 4, 66.6%) was the commonest bone involved.

**Figure 6:**
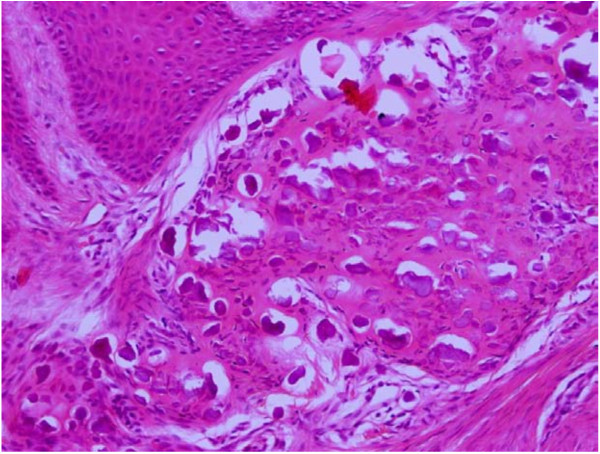
**Microphotograph of calcifying epithelial odontogenic tumor with calcified masses around epithelial cells**. H&E. 20×.

Calcifying epithelial odontogenic tumors (N = 5, 7.1%) (Figure 6) followed adenomatoid odontogenic tumors in frequency constituting the third most common type of the odontogenic tumors. Most of the patients presented in the early years of 11 to 20 and mandible was the commonly affected bone. Males were more commonly affected than the females.

Odontogenic myxomas constituted 4.3% (N = 3) of the odontogenic tumors (N = 70) with majority of the patients presenting between the ages of 21 and 30 years. Females outnumbered males (N = 2) in involvement with mandible being the more predominantly affected bone.

**Figure 7:**
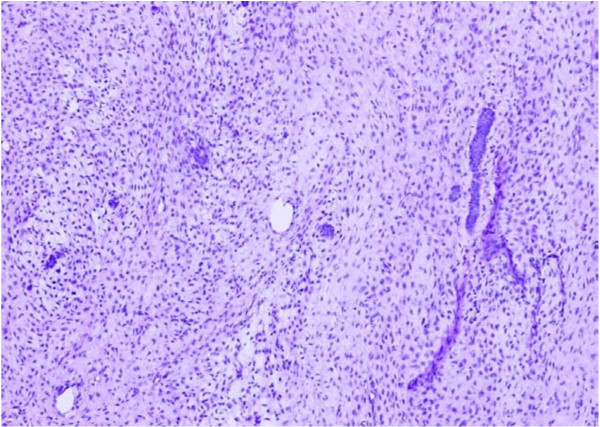
**Microphotograph of ameloblastic fibroma with odontogenic epithelium embedded in cellular mesodermal tissue**. H&E. 20×.

Odontogenic myxomas constituted 4.3% (N = 3) of the odontogenic tumors. These were followed in frequency by odontogenic fibromas constituting 2.8% (N = 2) of the odontogenic tumors and a single case (1.4%) of ameloblastic fibroma. (Figure 7)

### Giant cell lesions constituted 7 (09%) of the 77 jaw lesions

**Figure 8:**
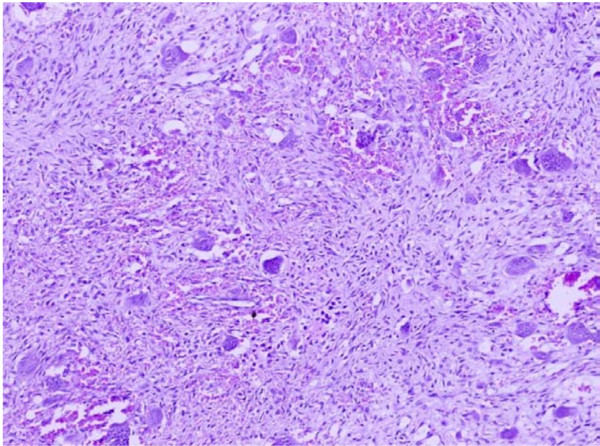
**Microphotograph of central giant cell granuloma showing vascular fibroblastic stroma and multinucleated giant cells**. H&E. 10×.

Among the 7 giant cell lesions encountered in our study, central giant cell granulomas (Figure 8) were the commonest in occurrence (N = 6, 7.8%) with majority of the patients (N = 3, 50%) presenting in the age group of 21 to 30 years. These lesions predominantly affected the mandible and females (N = 4, 66.6%).

Central giant cell granulomas were followed in frequency by a single case (1.2%, N = 1) of aneurysmal bone cyst.

## Discussion

A total of 77 patients with different tumors of the jaw bones presented in our institute between the years 2000 and 2009. The aim of our present study was to retrospectively analyze these varied lesions clinico-pathologically and to compare our data with the data from different parts of our country and also the world. Till date most of the studies reported on these lesions are mostly from America, Europe and Africa. Very few studies have been reported from Asia, especially from the Indian subcontinent. It has been observed that most of the Indian studies are either individual case reports or only a few limited series on these tumors [6-8,37,38]. Based on Indian literature survey, to date, we are herewith reporting for the first time a study of this nature as, it has been observed that only individual case studies have been reported in the Indian context.

Odontogenic tumors have a specific histological structure reflecting various stages of odontogenesis and are located mainly in the jaws, exceptionally in other parts of the skeletal system [3]. Due to their specific structure and location they have been identified and classified by pathologists into a separate group of neoplasms differing from other tumors developing in the oral cavity and facial bones [3]. While the giant cell lesions of the jaws are benign, tumor-like lesions affecting the jaws but also occurring in other bones and soft tissues.

In our study, all of the jaw tumors were reported as benign. Varkhede et al too, in their study, reported all their lesions as benign [8]. SrimmG and Shetty similarly reported 98.8% of the lesions as benign in their study[6]. Among these benign tumors, ameloblastomas were the most commonly encountered tumors which is in agreement with the previous reports by Srimm G & Shetty[6], Varkhede [8], Gupta[7], Ogunsalu[15], Adebayo[20, Stypulkowska[3], Fernandes[18] and Arotiba[19].

These varied jaw lesions have been observed to be more prevalent in the males than in females with most of the patients presenting in the 2^nd ^to the 3^rd ^decade of life. Majority of these lesions are located in the mandible [6-10]. However, Stypulkowska has reported a female preponderance (55.5%) of these lesions in his study [3] which is in contrast to the previous studies. The mean age of the patients in his study was however 32.5 years. In our study, these lesions were found to be more common in males (53.2%) than in females (46.8%). This contrasts the finding of female preponderance of these lesions by Stypulkowska. Figure 1 demonstrates the mean age of the patients in our study as 25 years which contrasts with the mean age of 32.5 years reported by Stypulkowska, including the studies from Ghana [9,10]. In addition, a few studies conducted in Nigeria have similarly reported a similar incidence of these lesions in both adults and children and mandible was the commonest jaw bone involved [11,12].

Most of these jaw lesions present with swelling, pain and ulceration [10]. In our study too most of these lesions commonly presented as slow-growing swellings. However, unlike the previous reports most were painless growths. Pain was observed in only 6 cases with none being ulcerated. Impacted tooth were found in 11 cases.

Radiologically, most of these jaw lesions appeared as well-defined, unilocular or multilocular, radiolucent lesions often associated with the crowns of impacted or unerupted teeth [13]. Our study too demonstrated majority of these jaw lesions (N = 58, 82.5%) as radiolucent with 36 among these (62%) being multilocular and 11 being associated with impacted teeth.

Table 1 demonstrates the frequency of distribution of the different odontogenic tumors and giant cell lesions of the jaws encountered in our study.

Ameloblastomas are benign epithelial neoplasms which develop from various sources of odontogenic epithelium, including dental follicular lining epithelium, and exhibit locally aggressive behavior [14]. The incidence of these tumors in different studies conducted till date showed a wide range from 11.7% to 73% [2,3,16-20]. The incidence of 71.4% (N = 50) reported in our study coincides with the incidence of 67% reported by Ogunsalu and Adebayo [15,20]. Similar to our study, ameloblastomas were most commonly encountered odontogenic tumors in studies by Srimm and Shetty [6], Gupta and Ponniah[7], Varkhede[8], Stypulkowska [3], Adebayo [15], Fernandes [18], Arotiba [19] and Ogansalu [20]. However, unlike our study, ameloblastomas were reported as second in incidence to odontomas in studies by Buchner et al [16], Mosqueda et al [17], Al-Khateeb [21], Tanaka [22] and Sato [23]. This finding proves that ameloblastomas are more commomly encountered tumors in Asians and Africans compared to Caucasians. Ameloblastomas manifest typically in the 3^rd ^to the 5^th ^decades of life [13,19,24]. In our study however most of the cases (N = 22) presented in a younger age group of 21 to 30 years. This finding of ours is similar to that reported by Reichart et al, who in their study stated that ameloblastomas occur in a younger age group in developing countries [25]. Most of the ameloblastomas present more in the mandible than maxilla [6-8,13,24,25]. In our study too, mandible (N = 43, 86%) was the more common site of presentation. Clinically most patients present with a slow growing painless mass [13,24,25] thus coinciding with finding of slow and painless growth of most of these lesions in our study. These tumors occur as expansile, radiolucent lesions which can be unilocular or multilocular, with a characteristic "soap bubble-like" appearance on radiology [24-26]. Our study too demonstrated majority of these lesions (N = 36, 72%) being multilocular on radiology.

Traditionally, ameloblastomas have been histologically divided into solid and cystic types [24]. One of the previous studies divided these tumors as one-third being plexiform, one-third follicular and other rare variants like acanthomatous, basaloid, desmoplastic and unicystic [25]. Hence, the two predominant patterns reported were follicular and plexiform types. We too in our study reported follicular pattern as the commonest (N = 25, 50%) followed by the plexiform type (N = 18, 40%). Figure 3 shows follicular ameloblastoma with epithelial islands. The central portion of these islands is composed of loose stroma while the outermost cells are tall columnar, with polarization of nuclei away from the basement membrane. Figure 2 shows the plexiform type with irregular masses and interdigitating cords of epithelial cells with minimal stroma.

Desmoplastic ameloblastoma is the most recently described microscopic pattern in which there is extensive desmoplasia and it is usually seen in the anterior jaws [27]. Figure 4 is the microphotograph of desmoplastic ameloblastoma in our study showing islands of odontogenic epithelium in a desmoplastic stroma.

Unicystic ameloblastoma was separated from the solid type because it appeared at an younger age and had a lower recurrence rate [28]. These tumors usually occur in the 2^nd ^to the 3^rd ^decades of life and in the mandibular molar area. Maxillary lesions are very uncommon [29]. In our study too, most of the unicystic ameloblastomas occurred in the age group of 11 to 20 years. However, due to the small number of cases of the above mentioned lesions (desmoplastic and unicystic ameloblastomas) in our study, we could not compare our results with previous reports.

Adenomatoid odontogenic tumor is a benign lesion that probably arises from the odontogenic epithelium of the dental lamina complex or its remnants [30-32]. Figure 5 shows the histology of this lesion which is composed of numerous ductal structures lined by cuboidal to tall columnar cells. It is more commonly seen in females, in the second decade of life with the most common location being the maxilla [8,24,26]. Although the tumor expands, it is not invasive and does not recur after conservative surgery. Similar to the incidence of 7.35 and 7.1% reported by Buchner et al [16] and Mosqueda et al [17] respectively, the incidence of these tumors reported in our study was 7.6%(N = 6), which was second in incidence to ameloblastomas. However, a lower incidence of 2% was reported by Stypulkowska [3] and Adebayo et al [12] in their studies which contrasts with the incidence of 7.6% observed in our study. The finding of greater incidence in women of the second decade in our study is similar to those of previous reports [6,8]. However, these tumors were more commonly located in the mandible. This finding contrasts with that of the earlier studies which reported maxilla as the more common site of occurrence the cause of which could be only explained by regional and geographic variation.

Calcifying epithelial odontogenic tumor or Pindborg's tumor is a rare odontogenic neoplasm of disputed histogenesis [33]. Figure 6 shows the histology of the tumor which is composed of closely packed polyhedral epithelial cells with a scant stroma. Numerous spherical spaces filled with eosinophilic homogenous material some of which is calcified are also seen. This tumor occurs more commonly in the fourth and fifth decades of life and in the same jaw sites (mandible) as of ameloblastoma [24,33]. It is a slow growing benign neoplasm and may be unilocular or multilocular [33]. Although described as a rare tumor, it was a relatively commonly encountered lesion (N = 5, 6.3%) in our study. The reported age incidence of 11 to 20 years in our study contrasts with the previous studies which reported these lesions as commonly occurring in the 4^th ^to the 5^th ^decades of life. Similar to the other studies mandible was the more commonly involved bone in our study.

An odontoma is an odontogenic hamartomatous malformation, often referred to as a tumor, which is composed of any or all odontogenic tissues in various states of morphological and histological differentiation [14]. Nearly 50% of odontomas are associated with an impacted tooth and most are diagnosed in the second decade of life [13,26]. Although reported as the commonest lesions by Robert et al [13], Buchner et al [16] and Mosqueda et al [17] in their studies, our study indicated a very low incidence of 3.8% (N = 3) of these lesions. Most odontomas do not produce symptoms clinically and are discovered incidentally on radiographs. This could be one of the reasons for a low incidence observed in our study as well as the Indian population because most patients in our country do not seek medical advice unless there are obvious clinical symptoms.

Odontogenic myxoma is an uncommon benign lesion constituting 3-6% of odontogenic tumors and originates from mesenchymal odontogenic tissue [13]. This tumor can be locally aggressive and cause considerable destruction of adjacent bone and soft tissue [14]. These tumors develop only in the bones of the jaws [34] and have a slight predilection for the maxilla [13]. However, Rosai has reported an equal incidence in mandible and maxilla [24]. Most of the previous studies have reported the age incidence as 10 to 30 years. Our incidence of 3.8% (N = 3) of these lesions is similar to that reported by Robert et al but is in contrast to a higher incidence of 9.2%, 17.7% and 9.1%, reported by Buchner et al [16], Mosqueda et al [17] and Fernandes et al [18] respectively. Although in our study 21 to 30 years was the commonest age of occurrence of these lesions, females outnumbered the males. Robert et al [13] too similarly reported a female preponderance of these lesions in their study. Mandible again was the more commonly involved of the jaw bones.

Two cases of odontogenic fibromas (N = 2, 2.5%) were reported in our study. Both the subjects were in the age group of 11 to 20 years. One case was male and the other was female. Manekar et al [38] reported 3 cases all of whom were females belonging to the age group of 15 to25 years.

Central giant cell granuloma appears to be lesion that is unique to the jaws. It was formerly regarded as reparative process and was accordingly called central giant cell reparative granuloma [35]. It is by far the most common of giant cell lesions of jaws and a history of trauma can often be ascertained [24]. It typically occurs more in females in the 2^nd ^and the 3^rd ^decades of life [13,24,26,33]. Mandible has been reported as more common jaw bone affected by this lesion [13,24,26,33]. Similar to the above studies, this lesion was the commonest of the 7 giant cell lesions reported in our study (N = 6, 85.7%) with a younger age of presentation of 21 to 30 years and females outnumbering males. Mandible similarly was the commonly involved bone. Two cases of central giant cell granulomas have been reported in the Indian literature [36,37] who were however both males aged 10 and 27 years. One presented with the lesion in mandible and the other in the maxilla. Radiologically, most of these lesions initially present as unilocular lesions which eventually become multilocular as they develop. Microscopically, it shows a large number of multinucleated giant cells, rather cellular vascular stroma and often new bone formation [24] as demonstrated in Figure 8.

A single case of aneurysmal bone cyst was encountered in our study. However, due to the paucity of these cases in our study, we could not compare our results with those of previous reports.

## Conclusion

In our present study, an attempt has been made to study the incidence of various odontogenic tumors and giant cell lesions of the jaws reported in our institute and to compare the results with reports from other parts of the world. Based on Indian literature survey to date we are herewith reporting for the first time a study of this nature from our region as it was observed that only few case studies have been reported in the Indian context.

We have demonstrated in our study that odontogenic tumours are not uncommon in our part of the world and there is a marked geographic variation in their relative incidences. Benign tumors are more common than malignant ones. Males are the more commonly affected gender than females and the age incidence peaked in the second decade of life, the cause of which probably could be related to the life style.

Radiology is seen to play an important role in the diagnosis of these lesions. Majority of the lesions had a predilection for the mandible and were multilocular radiolucent swellings. Clinically the majority presented as slow growing painless lumps indicating a benign nature of these lesions.

Histologically, ameloblastomas of the solid/microcystic type were the most commonly encountered tumors in our study while in contrast, odontomas were uncommon, the cause of which could be attributed to regional and geographical variations. Central giant cell granuloma was the most commonly encountered lesion in the giant cell category. The incidence of other tumors in our study was almost similar to those from other parts of the world.
